# Role of Parkin and endurance training on mitochondrial turnover in skeletal muscle

**DOI:** 10.1186/s13395-018-0157-y

**Published:** 2018-03-17

**Authors:** Chris Chin Wah Chen, Avigail T. Erlich, David A. Hood

**Affiliations:** 1 School of Kinesiology and Health Science, York University, Toronto, Ontario M3J 1P3 Canada; 2 Muscle Health Research Centre, York University, Toronto, Ontario M3J 1P3 Canada

**Keywords:** Endurance training, Mitochondrial biogenesis, Mitophagy flux, PGC-1α, PARIS

## Abstract

**Background:**

Parkin is a ubiquitin ligase that is involved in the selective removal of dysfunctional mitochondria. This process is termed mitophagy and can assist in mitochondrial quality control. Endurance training can produce adaptations in skeletal muscle toward a more oxidative phenotype, an outcome of enhanced mitochondrial biogenesis. It remains unknown whether Parkin-mediated mitophagy is involved in training-induced increases in mitochondrial content and function. Our purpose was to determine a role for Parkin in maintaining mitochondrial turnover in muscle, and its requirement in mediating mitochondrial biogenesis following endurance exercise training.

**Methods:**

Wild-type and Parkin knockout (KO) mice were trained for 6 weeks and then treated with colchicine or vehicle to evaluate the role of Parkin in mediating changes in mitochondrial content, function and acute exercise-induced mitophagy flux.

**Results:**

Our results indicate that Parkin is required for the basal maintenance of mitochondrial function. The absence of Parkin did not significantly alter mitophagy basally; however, acute exercise produced an elevation in mitophagy flux, a response that was Parkin-dependent. Mitochondrial content was increased following training in both genotypes, but this occurred without an induction of PGC-1α signaling in KO animals. Interestingly, the increased muscle mitochondrial content in response to training did not influence basal mitophagy flux, despite an enhanced expression and localization of Parkin to mitochondria in WT animals. Furthermore, exercise-induced mitophagy flux was attenuated with training in WT animals, suggesting a lower rate of mitochondrial degradation resulting from improved organelle quality with training. In contrast, training led to a higher mitochondrial content, but with persistent dysfunction, in KO animals. Thus, the lack of a rescue of mitochondrial dysfunction with training in the absence of Parkin is the likely reason for the impaired training-induced attenuation of mitophagy flux compared to WT animals.

**Conclusions:**

Our study demonstrates that Parkin is required for exercise-induced mitophagy flux. Exercise-induced mitophagy is reduced with training in muscle, likely due to attenuated signaling consequent to increased mitochondrial content and quality. Our data suggest that Parkin is essential for the maintenance of basal mitochondrial function, as well as for the accumulation of normally functioning mitochondria as a result of training adaptations in muscle.

## Background

Skeletal muscle is a highly malleable tissue that displays a remarkable adaptive plasticity. Chronic contractile activity of muscle can evoke molecular and biochemical adaptations that enable muscle to improve its oxidative capacity [1, 2]. This enhancement is, in part, the result of greater mitochondrial enzyme gene expression [3, 4]. It is well established that endurance training can increase mitochondrial biogenesis and alter muscle metabolism [5–7]. In trained muscle, an elevation in mitochondrial content improves oxidative phosphorylation, while lowering the reliance on glycolysis [8, 9]. The process of generating new organelles is mediated, in part, by the transcriptional regulator peroxisome proliferator-activated receptor-γ coactivator-1α, PGC-1α. During acute bouts of exercise, PGC-1α can translocate into the nucleus [6, 10, 11] and upregulate the transcription of nuclear genes encoding mitochondrial proteins (NUGEMPs) [12], as well as transcription factors [13, 14] such as mitochondrial transcription factor A (TFAM). TFAM is a nuclear-encoded protein involved in mitochondrial DNA (mtDNA) transcription, along with the expression of several protein subunits of the respiratory electron transport chain [15]. Chronic contractile activity can increase TFAM protein expression, followed by accelerated mitochondrial protein import and mtDNA binding [16]. TFAM abundance has been shown to increase in response to training in humans [17]. Thus, the induction of mitochondrial biogenesis during exercise requires the coordination of both nuclear and mitochondrial genomes for subsequent organelle assembly and function [18–20].

Mitochondria are considered to be primary sites for free radical formation [21, 22]. When this production is left unchecked, oxidative stress can impair ATP generation and induce cellular injury. To combat against sustained damage, the selective removal of dysfunctional mitochondria, known as mitophagy, is swiftly activated. Organelle turnover is the balance between mitochondrial biogenesis and mitophagy, and the combination of these processes aids in the maintenance of energetic homeostasis. An imbalance of these two opposing pathways can lead to the development of cardiovascular diseases [23, 24], cancer [25, 26], and diabetes [27, 28] and accelerate aging [29–31]. The most well characterized pathway of mitophagy involves PTEN-induced putative kinase 1 (PINK1) and ubiquitin ligase Parkin [32]. Under non-stressful conditions, PINK1 import into polarized mitochondria ensues and is followed by its proteolysis [33]. During cellular perturbations in which a collapse in organelle membrane potential occurs, PINK1 import into the matrix is impaired and its stabilization on the outer mitochondrial membrane (OMM) serve to recruit and activate Parkin [34–36]. PINK-1 mediated phosphorylation of Parkin [37, 38] and ubiquitin [39–41] allows for the accretion of polyubiquitin chains on several protein targets of the OMM. This mobilizes the autophagy adaptor protein p62 to tagged mitochondria through its ubiquitin-binding domain [42, 43]. Lipidated microtubule-associated protein-light chain 3 (LC3II) is a protein bound on the phagophore membrane that can associate with p62 [44, 45], ultimately resulting in the autophagosomal enclosure of dysfunctional mitochondria as a cellular safeguard from additional oxidative damage [46]. The autophagosome is then terminally degraded at the lysosome.

Recent evidence suggests that acute exercise is sufficient to activate autophagy [47, 48]. However, few studies have examined the potential role of mitophagy during exercise. We have previously shown that skeletal muscle of exercised mice exhibits enhanced mitophagy flux, and that this is partially dependent on PGC-1α [11]. In another study, the acute inhibition of autophagy in mice led to an accumulation of dysfunctional mitochondria in locomotor muscles during damaging eccentric exercise [49]. While these studies collectively demonstrate that mitochondrial degradation occurs during acute exercise, they do not clarify the effects of prior endurance training and how it can influence mitophagy. Current research seems to propose that autophagy may be enhanced in endurance-trained muscle as an adaptation in improving exercise performance [50–53]. Yet, these studies do not provide a direct measurement of mitophagy using flux measurements nor do they indicate how mitophagy may interact with the process of biogenesis that occurs with endurance training. In particular, Parkin has been shown to regulate both mitochondrial biogenesis and mitophagy, and the in vitro overexpression of wild-type Parkin can interact with TFAM to increase mtDNA transcription and replication, as well as increase mitochondrial mass [54, 55]. Parkin can also increase PGC-1α protein expression through downregulation of Parkin-interacting substrate (PARIS) [56, 57]. The activation of Parkin can target PARIS, a transcriptional repressor of PGC-1α, to the proteasome for degradation [58]. Thus, Parkin may serve as a potential channel of communication between mitophagy and mitochondrial biogenesis. The purpose of our study was to investigate how Parkin is involved in mediating both biogenesis and mitophagy using WT and Parkin KO mice that were either sedentary or subjected to endurance training via voluntary wheel running. We also examined mitophagy flux in trained animals with a subsequent bout of acute exercise to determine the adaptive effect of prior endurance training.

## Methods

### Animal model

C57BL/6 (WT) and B6.129S4-Park2^tm1Shn^/J (Parkin KO; 006582) mice were obtained from Jackson Laboratories. The generation of these mice has been previously described [59]. To genotype progeny, ear clippings were obtained from each animal for DNA extraction. JumpStart REDtaq polymerase (Sigma-Aldrich, St. Louis, MO) was incubated with DNA extracts, as well as forward and reverse primers specific to nucleotides of the WT or altered Parkin gene, and amplified using PCR. The reaction products were separated on a 1.5% agarose gel and visualized with the use of ethidium bromide.

### Voluntary wheel running

Three-month-old WT and Parkin KO mice were assigned to control or trained experimental groups. During the duration of the study, all mice were housed in a 12-h light–dark cycle room, and were allowed access to water and food ad libitum. Runners were placed in cages with access to a freely rotating wheel attached to an external magnetic counter (Mini-Mitter, Bend, OR). The number of revolutions was noted every 24 h for each animal and converted into kilometers per week. The duration of the training protocol lasted 6 weeks.

### Endurance exercise protocol and blood lactate measurement

Following the training protocol, cage wheels were taken out 2 days prior to treadmill habituation and acute exercise. During these 2 days, animals were injected intraperitoneally with colchicine (0.4 mg kg^− 1^ day^− 1^), or an equal amount of vehicle (water) every 24 h [60]. Injections were performed before mice were acclimatized to the treadmill. Vehicle- and colchicine-treated animals in the exercise (Ex) group ran on a fixed, upward treadmill slope of 10°. Mice ran at 5 m/min for 5 min, 10 m/min for 10 min, 15 m/min for 15 min, and 20 m/min for 20 min. The speed was then incrementally increased at 1 m/min for every 1 min until exhaustion was achieved. Exhaustion was defined as the inability of the animal to continue running on the treadmill, even in the presence of air jet stimulation. A small tail bleed was used to measure blood lactate with a Lactate Scout+ analyzer (EKF Diagnostics, Magdeburg, Germany). All animals were sacrificed by cervical dislocation immediately after exercise (Ex).

### Cytochrome c oxidase (COX) enzyme activity

COX activity was measured as previously detailed [61] and performed on untrained and trained groups of WT and Parkin KO mice to measure whole muscle mitochondrial content. Briefly, protein extracts from mixed hindlimb muscles were added to a test solution containing fully reduced cytochrome c. Enzyme activity was determined by the maximal oxidation rate of completely reduced cytochrome c, measured by the change in absorbance at 550 nm using a Bio-Tek Synergy HT microplate reader, as previously described [6].

### Mitochondrial isolation

Mixed hindlimb muscles from both sides of the animal were immediately placed into ice-cold mitochondrial isolation buffer and were quickly minced and homogenized. Intermyofibrillar (IMF) mitochondrial sub-fractions were subjected to differential centrifugation, as described previously [6, 62, 63]. Mitochondria were resuspended in 100 mM KCl, 10 mM MOPS, and 0.2% BSA. Freshly isolated mitochondria were used for mitochondrial respiration and reactive oxygen species (ROS) emission assays, and aliquots of mitochondrial extracts were stored at − 80 °C for immunoblotting analyses. The protein concentration values of the isolated mitochondria were quantified using the Bradford method.

### Mitochondrial respiration

Samples of isolated IMF mitochondria were incubated with VO_2_ buffer (in mM: 250 sucrose, 50 KCl, 25 Tris, and 10 K2HPO4, pH 7.4) with continuous stirring in a respiratory chamber. Respiration rates (nanoatom of O_2_/min/mg) were driven by complex I of the mitochondrial electron transport chain in the presence of 10 mM glutamate (state 4, Sigma) accompanied by the addition of 0.44 mM ADP (state 3, Sigma). The addition of NADH during state 3 respiration did not significantly alter respiration rates, indicating intact inner mitochondrial membrane integrity. All respiration rates were evaluated using the Mitocell S200 Micro Respirometry System (Strathkelvin Instruments, North Lanarkshire, UK).

### Mitochondrial ROS production assay

ROS emission was measured as done previously [6, 64]. Briefly, isolated IMF mitochondria were incubated with VO_2_ buffer at 37 °C for 30 min in a white polystyrene 96-well plate under state 4 and 3 conditions. The addition and oxidation of 2′, 7′-dichlorodihydrofluorescein diacetate (50 μM H2DCFDA, Thermo Fisher) emitted fluorescence between 480 and 520 nm, as measured with a Bio-Tek Synergy HT microplate reader, and is directly related to ROS emission. ROS emission was normalized per nanoatom of O_2_ consumed, as measured during mitochondrial respiration.

### Immunoblotting

Whole quadricep muscle extracts were performed as described previously [64]. Briefly, frozen muscle samples were pulverized, resuspended in buffer, sonicated and centrifuged. Muscle lysate was attained in the supernatant fraction, and the Bradford technique was used to determine protein content. Muscle homogenates and isolated mitochondrial fractions were separated using SDS-PAGE (12–15% polyacrylamide) and transferred to nitrocellulose membranes (BioRad). Blots were incubated overnight at 4 °C with primary antibodies against LC3/microtubule-associated protein light chain 3 (4108, Lot No. 3, Cell Signaling), p62/sequestosome 1 (P0067, Lot No. 015M4877V, Sigma), Parkin (4211, Lot No. 4, Cell Signaling), PGC-1α/peroxisome proliferator-activated receptor-γ coactivator-1α (ab3242, Lot No. 2691399, Millipore), PARIS/Parkin-interacting substrate (ab130867, Lot No. GR235090-1, abcam), phospho-acetyl-CoA carboxylase (07-303, Lot No. DAM1661071, Millipore), COXI/cytochrome c oxidase subunit 1 (ab14705, Lot No. GR233531-3, Abcam), COXII/cytochrome c oxidase subunit 2 (bs-2376R, Lot No. YEYL31W, Bioss), COXIV/cytochrome c oxidase subunit 4 (ab140643, Lot No. GR192963-3, Abcam), TFAM/mitochondrial transcription factor A (in house), VDAC/voltage-dependent anion channel (ab14734, Lot No. GR121056-7, Abcam), GAPDH (ab8245, Lot No. GR3185172-2, Abcam), and α-tubulin (CP06, Lot No. D00175772, Millipore). This was followed by an hour incubation period at room temperature with appropriate horseradish peroxidase-conjugated secondary antibodies. The membranes were visually distinguished using enhanced chemiluminescence (Clarity ECL Western blotting substrates, Bio-Rad, CA) and photographic film. Films were quantified using ImageJ software (Version 1.48, NIH, USA).

### Statistical analysis

Data were analyzed with Graph Pad 6.0 software, and values are reported as means ± SEM. Data were analyzed using two-way analysis of variance (ANOVA), except for Fig. 2a which was analyzed using Student’s *t* test. For all two-way ANOVA analyses, Tukey’s post hoc test was used to identify individual differences when statistical significance was observed. Statistical differences were considered significant if *P* < 0.05. All N values in the manuscript represent total animals used per condition.

## Results

### Physical characteristics of Parkin KO mice following endurance training

**Fig. 1:**
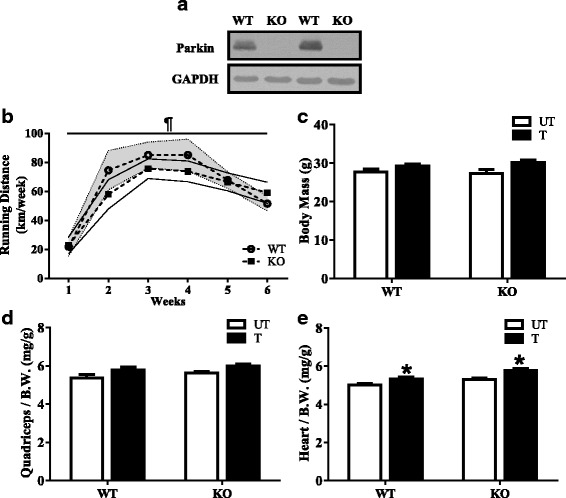
Effect of Parkin deficiency and training on muscle mass. **a** Representative Western blot of Parkin expression in skeletal muscle of control wild-type (WT) and Parkin knockout (KO) mice. **b** Graphical representation of voluntary running distance (kilometers per week) between WT and KO mice (*n* = 8). Wheels were provided in animal cages for a duration of 6 weeks. **c** Total body weight of untrained and trained Parkin KO and WT animals (*n* = 8). **d** Quadriceps muscle mass corrected for total body weight (*n* = 8). **e** Heart mass corrected for total body weight (*n* = 8). Values are means ± SEM. ¶*P* < 0.05, main effect of time. *P < 0.05, main effect of training. WT, wild type; KO, Parkin knockout; UT, untrained; T, trained

To determine the physiological importance of Parkin with training in skeletal muscle, we first assessed the physical characteristics of untrained and trained groups of WT and Parkin KO mice. During the 6 weeks of voluntary wheel running, WT and Parkin KO mice did not significantly differ in running distance, with both genotypes running up to ~ 70 km/week (*P* < 0.05; Fig. 1b). Total body mass, quadriceps, and heart weight were measured at the end of the 6 weeks of voluntary exercise. There was no significant effect of genotype or training on body mass (Fig. 1c) or quadriceps muscle mass when corrected for body weight (Fig. 1d). In addition, no differences between WT and KO animals existed with respect to heart mass when corrected for body weight, but both genotypes exhibited ~ 5–7% elevations following training (*P* < 0.05; Fig. 1e).

### The expression of several mitochondrial markers is promoted following 6 weeks of voluntary exercise training

**Fig. 2:**
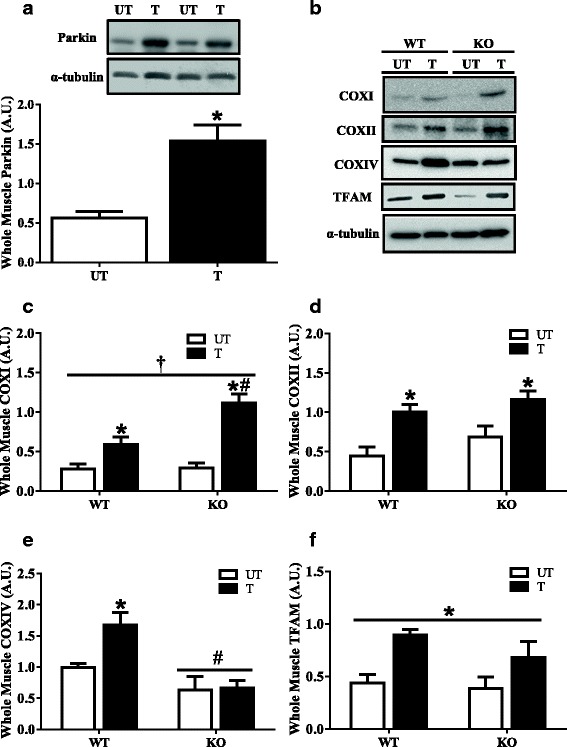
Effect of training and Parkin on whole muscle protein markers. **a** Representative Western blot of Parkin expression in untrained and trained skeletal muscle of control wild-type (WT) mice above. A graphical representation is shown below (*n* = 5, *P < 0.05, vs UT). **b** Representative immunoblots of COXI, COXII, COXIV and TFAM expression in untrained and trained skeletal muscle of Parkin KO and WT animals. **c** A graphical representation of whole muscle COXI shown (*n* = 4, *P < 0.05, main effect of training; #*P* < 0.05, vs trained WT). **d** Quantification of whole muscle COXII (*n* = 6, *P < 0.05, main effect of training). **e** Whole muscle COXIV expression (*n* = 4, *P < 0.05, main effect of training, #*P* < 0.05, main effect of genotype). **f** Quantification of whole muscle TFAM (*n* = 4, *P < 0.05, main effect of training). Values are means ± SEM. **†***P* < 0.05, interaction effect of training and genotype. α-tubulin was used for muscle loading control. WT, wild type; KO, Parkin knockout; UT, untrained; T, trained; COXI, cytochrome c oxidase subunit 1; COXII, cytochrome c oxidase subunit 2; COXIV, cytochrome c oxidase subunit 4; TFAM, mitochondrial transcription factor A; A.U., arbitrary units

We examined the influence of endurance training on the whole muscle expression of several protein markers involved in mitochondrial turnover. A large ~ 3-fold increase in Parkin expression was observed in trained compared to untrained muscle (*P* < 0.05; Fig. 2a). A significant interaction of genotype and training was observed for COXI (*P* < 0.05; Fig. 2b, c). Basal COXI expression did not differ between genotypes, but COXI expression was elevated by 2.1- and 3.8-fold in trained WT and Parkin KO animals, respectively, when compared to the untrained cohort (*P* < 0.05; Fig. 2b, c). COXII expression did not differ between genotypes, but displayed similar 1.7–2.2-fold increases following training (*P* < 0.05; Fig. 2b, d). In contrast, COXIV expression was elevated by 1.6-fold by training, but only in WT animals (Fig. 2b, e). Whole muscle COXIV levels were significantly depressed in the absence of Parkin, even with endurance training (Fig. 2b, e). Thus, the levels of COX nuclear and mitochondrial subunits were differentially affected by training, in a Parkin-dependent manner. TFAM protein expression did not differ between genotypes (Fig. 2b, f). However, training induced a significant ~ 2-fold increase in TFAM expression in both WT and KO animals.

**Fig. 3:**
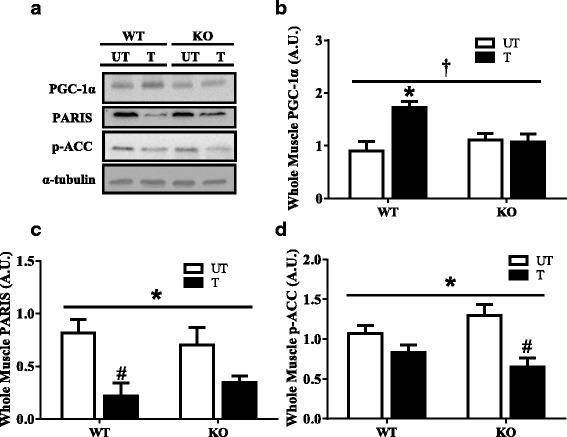
Effect of training and Parkin on key regulators of mitochondrial biogenesis. **a** Representative Western blots of PGC-1α, PARIS and p-ACC expression in untrained and trained skeletal muscle of Parkin KO and WT animals. **b** A graphical representation of whole muscle PGC-1α (*n* = 4, *P < 0.05, vs untrained WT). **c** Quantification of whole muscle PARIS (*n* = 6, *P < 0.05, main effect of training; #*P* < 0.05, vs untrained WT). **d** A graphical representation of whole muscle phosphorylation of ACC (*n* = 6, *P < 0.05, main effect of training; #*P* < 0.05, vs untrained KO). Values are means ± SEM. **†***P* < 0.05, interaction effect of training and genotype. α-tubulin was used for muscle loading control. WT, wild type; KO, Parkin knockout; UT, untrained; T, trained; p-ACC, phospho-acetyl-CoA carboxylase; PARIS, Parkin-interacting substrate; PGC-1α, peroxisome proliferator gamma coactivator-1α; A.U., arbitrary units

Parkin can regulate mitochondrial biogenesis by targeting Parkin-interacting substrate (PARIS) to the proteasome for degradation. In the absence of Parkin, PARIS can transcriptionally repress PGC-1α, a transcriptional co-activator of mitochondrial biogenesis. Surprisingly, whole muscle PGC-1α and PARIS protein expression did not differ between untrained WT and Parkin KO animals (Fig. 3a–c). Rather, a significant interaction of training and genotype for PGC-1α emerged (*P* < 0.05; Fig. 3b). Training induced a 1.9-fold increase in whole muscle PGC-1α expression in WT animals that was abolished in the absence of Parkin (*P* < 0.05; Fig. 3a, b). Similarly, a main effect of training on whole muscle PARIS levels was observed in both genotypes. Most notable was the large 75% reduction in WT animals (*P* < 0.05; Fig. 3a, c). AMPK is an upstream regulator of both PGC-1α and its downstream target acetyl-CoA carboxylase (ACC). We measured ACC as an indicator of basal AMPK activity, and a significant main effect of training on the suppression of phosphorylated ACC was detected (*P* < 0.05; Fig. 3a, d). While this reduction was relatively minor in WT animals (25%), the KO animals displayed a significant 50% reduction in phosphorylated ACC in trained compared to untrained mice (*P* < 0.05; Fig. 3a, d).

### Mitochondrial adaptations in Parkin KO animals following exercise training

**Fig. 4:**
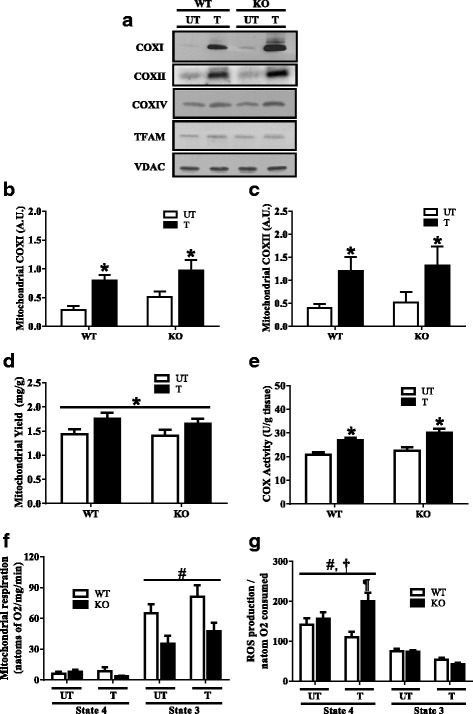
Mitochondrial adaptations following 6 weeks of voluntary wheel training. **a** Representative Western blots of COXI, COXII, COXIV and TFAM expression on isolated mitochondria from untrained and trained skeletal muscle of Parkin KO and WT animals. **b** Graphical representation of mitochondrial COXI expression (*n* = 8). **c** Quantification of mitochondrial COXII content (*n* = 4). **d** Mitochondrial yield of mixed hindlimb muscles of Parkin KO and WT animals (*n* = 4). **e** Skeletal muscle cytochrome c oxidase (COX) activity following training in quadriceps muscle of Parkin KO and WT animals (*n* = 5). **f** Mitochondrial state 4 and state 3 respiration rates in KO compared with WT animals (*n* = 8). **g** Mitochondrial ROS emission expressed per natom of oxygen consumed in Parkin KO and WT mice (*n* = 8; ¶*P* < 0.05, vs state 4 trained WT). Values are means ± SEM. *P < 0.05, main effect of training; #*P* < 0.05, main effect of genotype; **†***P* < 0.05, interaction effect of genotype and training. Voltage-dependent anion channel (VDAC) was used as a mitochondrial loading control. WT, wild type; KO, Parkin knockout; UT, untrained; T, trained; COX1, cytochrome c oxidase subunit 1; COXII, cytochrome c oxidase subunit 2; COX4, cytochrome c oxidase subunit 4; TFAM, mitochondrial transcription factor A; ROS, reactive oxygen species; A.U., arbitrary units

We next evaluated the effects of Parkin deficiency and exercise training on muscle mitochondrial protein composition, yield and function. Interestingly, the expression of nuclear-encoded mitochondrial proteins COXIV and TFAM did not differ between genotypes, or with training (Fig. 4a), despite significant changes at the whole muscle level (Fig. 2). In contrast, mitochondrially encoded subunits COX I and II did not differ between genotypes, but exhibited significant two- to threefold increases following 6 weeks of exercise training (*P* < 0.05; Fig. 4a–c), paralleling protein changes evident in whole muscle extracts (Fig. 2) in both genotypes. Mitochondrial yield represents the amount of mitochondrial protein extracted corrected for the total muscle weight used for the mitochondrial isolation. Although there was no difference between untrained WT and KO animals, both genotypes displayed significant increases with endurance training (*P* < 0.05; Fig. 4d). The biochemical assessment of cytochrome c oxidase (COX) activity confirmed the lack of difference between genotypes with respect to an enzymatic marker of muscle mitochondrial content. In addition, exercise training significantly increased COX activity by ~ 1.4-fold in both WT and Parkin KO mice (*P* < 0.05; Fig. 4e). We also examined whether mitochondrial respiration and reactive oxygen species (ROS) emission differed with voluntary wheel running, and in the absence of Parkin. No significant training effect was noted on mitochondrial respiration. However, a main effect of genotype was observed on state 3 mitochondrial respiration (*P* < 0.05; Fig. 4f). When untrained animals were compared, a significant 46% decrease in state 3 mitochondrial respiration was detected in KO, compared to WT mice (*P* < 0.05; Fig. 4f). Following endurance training, the impairment in organelle respiration remained in KO animals (*P* < 0.05; Fig. 4f). Similarly, a significant main effect of genotype was noted on mitochondrial ROS emission (*P* < 0.05; Fig. 4g) during state 4 respiration, and a significant interaction between genotype and the training response was observed. Whereas ROS emission tended to decline with training in WT animals, a significant 1.8-fold difference in ROS emission was noted between trained KO and WT animals (*P* < 0.05; Fig. 4g). These respiration and ROS data suggest that Parkin is required for the restorative effect of exercise training on mitochondrial function.

### Endurance training results in improved exercise performance and attenuated mitochondrial Parkin localization following acute exercise

**Fig. 5:**
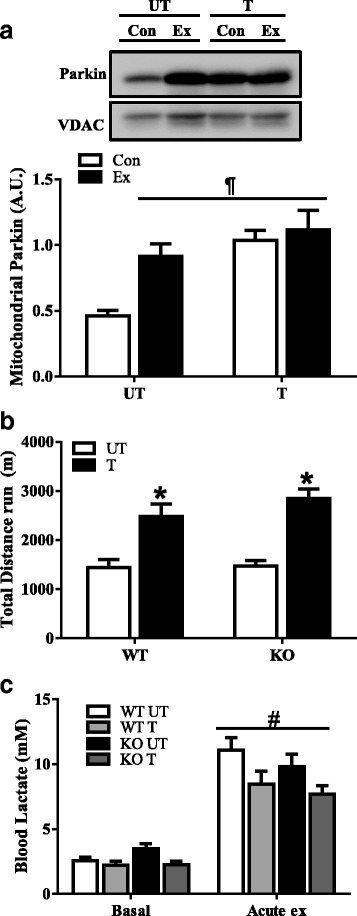
Effect of Parkin and training on acute exercise performance. **a** Representative Western blot of Parkin localization on isolated mitochondria from untrained and trained WT muscle, prior to exercise (Con) and immediately following exercise (Ex). Quantification of mitochondrial Parkin localization is shown below, corrected for loading using mitochondrial voltage-dependent anion channel (VDAC) (*n* = 6, ¶*P* < 0.05, vs untrained Con). **b** Animal endurance performance (i.e. total distance run) of WT and KO animals (*n* = 8, *P < 0.05, main effect of training). **c** Blood lactate levels measured prior to (Con), and immediately following exercise (Ex) (*n* = 8, #*P* < 0.05, main effect of exercise). Values are means ± SEM. WT, wild type; KO, Parkin knockout; UT, untrained; T, trained; A.U., arbitrary units

Following the 6 weeks of voluntary wheel training, we assessed the adaptive response of the muscle to an acute bout of exercise. In untrained WT animals, we found a twofold increase in Parkin localization to the mitochondria immediately following acute exercise (*P* < 0.05; Fig. 5a). In trained animals, organelle Parkin localization was already high under resting, control condition, and did not increase additionally with an acute bout of exercise (*P* < 0.05; Fig. 5a). Untrained WT and Parkin KO animals did not differ in their endurance performance when subjected to a bout of acute exercise (Fig. 5b). However, with training, both genotypes displayed ~ 1.8-fold increases in running distances when compared to their untrained counterparts (*P* < 0.05; Fig. 5b). Resting blood lactate levels were at ~ 2 mM, and significantly rose to ~ 7–11 mM immediately after acute exercise (*P* < 0.05; Fig. 5c). There was no genotype effect on blood lactate levels after exercise, and a trend was observed for reduced lactate concentrations following training.

### Mitophagy flux is induced following exercise, but this signaling is attenuated in the absence of Parkin, and with training

**Fig. 6:**
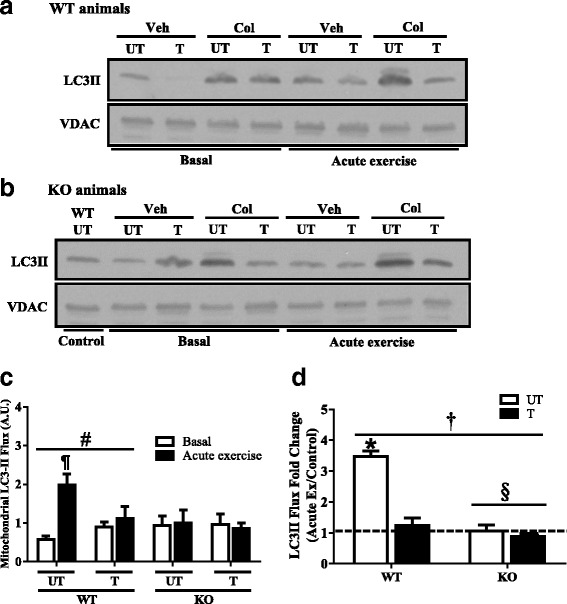
Mitophagic LC3II flux following acute exercise, training, and combined treatments in WT and KO animals. Representative Western blots of mitochondrial LC3II localization of WT (**a**) and Parkin KO (**b**) mice injected with water (Veh) or 0.4 mg/kg colchicine (Col). Quantification of mitochondrial LC3II flux (**c**) is shown (*n* = 6). Mitophagic LC3II flux was assessed under basal conditions, and immediately following acute exercise, in untrained and trained groups of WT and KO animals. The fold change in LC3II flux (*D*) was calculated with acute exercise-induced values over basal values (*n* = 6). Values are means ± SEM. ¶*P* < 0.05, vs untrained WT; #*P* < 0.05, main effect of exercise; §*P* < 0.05, main effect of genotype; *P < 0.05, vs all other experimental conditions; **†***P* < 0.05, interaction effect of genotype and training. Voltage-dependent anion channel (VDAC) was used as a mitochondrial loading control. WT, wild type; KO, Parkin knockout; UT, untrained; T, trained; LC3II, lipidated microtubule-associated protein 1A/1B-light chain 3; A.U., arbitrary units

**Fig. 7:**
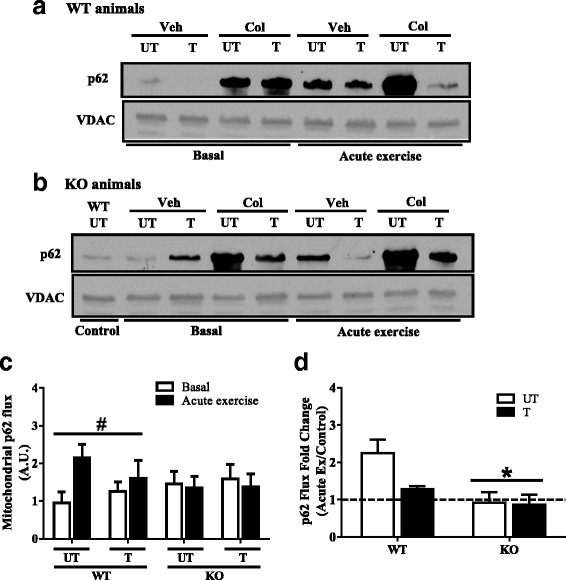
Mitophagic p62 flux following acute exercise, training, and combined treatments in WT and KO animals. Representative Western blots of mitochondrial p62 localization of WT (**a**) and Parkin KO (**b**) mice injected with water (Veh) or 0.4 mg/kg colchicine (Col). Quantification of mitochondrial p62 flux (**c**) is shown (*n* = 5). Mitophagic p62 flux was assessed under basal conditions, and immediately following acute exercise, in untrained and trained groups of WT and KO animals. The fold change in p62 flux (**d**) was calculated with acute exercise-induced values over basal values (*n* = 5). Values are means ± SEM. *P < 0.05, main effect of genotype; #*P* < 0.05, main effect of exercise. Voltage-dependent anion channel (VDAC) was used as a mitochondrial loading control. WT, wild type; KO, Parkin knockout; UT, untrained; T, trained; p62, sequestosome 1; A.U., arbitrary units

To determine a role for Parkin in mediating training-induced changes in exercise-induced mitophagy flux, mice were subjected to 6 weeks of voluntary wheel training, followed by a bout of acute exercise. Autophagy protein localization was quantified on isolated mitochondria from the hindlimb muscles of mice for each condition. Immunoblots were separated by genotypes due to the number of conditions. We normalized each experimental condition to an untrained WT sample, from the same data set, to determine any differences between genotypes (Figs. 6a, b and 7a, b). Following this, we calculated autophagy flux based on the difference between colchicine- and vehicle-treated animals. When untrained and trained animals were compared, no differences in basal LC3II and p62 flux were detected. In WT mice only, a main effect of acute exercise on LC3II and p62 flux was observed between untrained and trained groups (*P* < 0.05; Fig. 6c and Fig. 7c). During acute exercise, robust 3.5- and 2-fold increases in mitochondrial LC3II and p62 flux, respectively, were measured in untrained WT animals (*P* < 0.05; Fig. 6c). This response was attenuated with voluntary wheel training.

To directly determine if Parkin was required for mediating these training-induced adaptations, we then compared mitophagy flux between WT and KO animals. In untrained animals, basal LC3II and p62 flux was not significantly different between genotypes (Fig. 6a–c and Fig. 7a–c). In contrast to WT animals, LC3II and p62 flux did not increase with acute exercise in the absence of Parkin. Similarly, basal LC3II and p62 flux was not altered in KO animals with 6 weeks of voluntary wheel training, or with a subsequent bout of endurance exercise.

To further analyze the adaptive potential of trained muscle, we calculated the fold change of mitophagy flux as a ratio of acute exercise-induced values over basal values. The analysis revealed a significant interaction of genotype and training on the fold change in LC3II flux (*P* < 0.05; Fig. 6d). Untrained WT animals displayed a 2.4-fold acute exercise-induced increase in LC3II flux, and this response was attenuated by 45% with training (Fig. 6d). As noted above, LC3II flux did not change with acute exercise in untrained and trained KO animals. In contrast to LC3II, a main effect of genotype was found when the fold change for p62 flux was calculated (*P* < 0.05; Fig. 7d). An increase in p62 flux was observed following a bout of endurance exercise in untrained WT animals. Following 6 weeks of voluntary wheel running, this response was attenuated by 43% (Fig. 7d). In the absence of Parkin, p62 flux remain depressed with acute exercise, and by training (Fig. 7d).

## Discussion

Endurance exercise training is accompanied by an adaptive increase in muscle oxidative capacity. To produce this beneficial phenotype, successive bouts of acute exercise over 6 to 8 weeks are required to elicit gradual, 50 to 100% elevations in mitochondrial protein content and enzyme activities [65]. Prior to attaining an elevated mitochondrial content, a temporal sequence of transcriptional, translational, and post-translational events must occur. However, the precise mechanisms leading to this adaptive response are not fully established [66]. Parkin is an E3 ubiquitin ligase that is known to be involved in the clearance of defective mitochondria for lysosomal degradation [67]. However, recent work has also indicated a possible role for Parkin in mitochondrial biogenesis. For example, in vitro overexpression of Parkin can associate with the regulatory protein TFAM and enhance TFAM-mediated mtDNA transcription [54, 55]. Furthermore, Parkin can directly target PARIS, a transcriptional repressor of PGC-1α, to the proteasome for degradation [56, 57], thereby promoting PGC-1α action on organelle biogenesis. Whether Parkin has a role in determining muscle function and adaptation to exercise training remains currently undetermined.

To investigate this, we examined the effect of voluntary wheel running for 6 weeks in WT and Parkin KO mice. In WT animals, we found that this training resulted in several expected beneficial consequences, such as improved running endurance performance, increases in markers of mitochondrial biogenesis, and modest cardiac hypertrophy. Interestingly, a robust increase in Parkin expression was also measured in WT animals following 6 weeks of training. We hypothesized that this increase in Parkin would enhance PARIS degradation and thereby promote PGC-1α expression [56]. As expected, we observed a significant increase in PGC-1α protein, accompanied by reduced PARIS expression following training in WT animals. These changes likely contributed significantly to the training-induced increases in mitochondrial content and composition at the organelle level, observed as a result of training.

Our research was directed to evaluate the influence of Parkin on the regulation of muscle mitochondrial content based, in part, on recent work suggesting that Parkin can mediate both organelle biogenesis [57, 58] and degradation [67]. Research in neuroblastoma cells and tissues has indicated that a deficiency in Parkin can enhance the repressive behavior of PARIS on PGC-1α transcription [56]. This could lead to a progressive impairment of mitochondrial biogenesis in a Parkin-dependent manner. However, our findings in skeletal muscle indicate that KO animals did not display a difference in mitochondrial content when compared to WT littermates. Moreover, PARIS and PGC-1α expression in muscle did not vary between genotypes. Thus, a role for Parkin in maintaining mitochondrial content via this mechanism seems unlikely, at least in muscle. Alternatively, Parkin is mainly implicated in organelle degradation as a ubiquitin ligase accountable for the removal of defective mitochondria. Thus, the absence of Parkin could lower the rate of mitophagy and increase the accumulation of dysfunctional organelles [68, 69]. Consistent with this, we found that KO mice exhibited reduced glutamate-ADP-stimulated state mitochondrial respiration, a result that was found in previous studies [70]. Thus, in contrast to the lack of difference in mitochondrial content between genotypes, our data provide evidence that the absence of Parkin can evoke mitochondrial dysfunction, as we have previously shown [11]. This is also consistent with a previously documented role for Parkin in the elimination of mitochondria with deleterious mtDNA mutations [71] and further suggests that Parkin is useful for the maintenance of normal, healthy mitochondrial pool in muscle.

We next sought to ascertain the role of Parkin in mediating exercise training-induced mitochondrial biogenesis in muscle. Our results clearly indicate that the absence of Parkin does not impede the inducing effect of exercise training on mitochondrial content, as evident from increases in whole muscle COX enzyme activity, as well as mitochondrially encoded COXI and COXII subunit protein expression. Surprisingly, these training effects occurred in the absence of any induction of PGC-1α and the expression of downstream nuclear-encoded COXIV subunit in trained KO animals, suggesting the presence of alternative signaling mechanisms involved in mitochondrial biogenesis in the absence of Parkin. This lack of PGC-1α induction with training was not likely accounted for by an increase in PARIS-mediated PGC-1α transcriptional repression, since training served to reduce PARIS expression. Our data illustrate that training either serves to reduce the synthesis of PARIS or to enhance its proteasomal degradation, even in the absence of Parkin-directed ubiquitination. Further, organelle biogenesis functions in concert with degradation to maintain mitochondrial content. The molecular events that are involved in mitochondrial degradation with either acute or chronic exercise remain poorly defined. It is hypothesized that acute exercise induces mitophagy as a cellular strategy of purging superfluous mitochondria [49]. A general challenge of studying autophagy is accurately measuring “flux”, an outcome of autophagosomal formation and degradation. In our study, we utilized microtubule destabilization drug colchicine, which prevents autophagosome-lysosome fusion [60]. We have previously provided evidence that mitophagy flux is enhanced following a single bout of endurance exercise [11], a finding that was replicated in this study. We detected significant mitochondrial Parkin localization immediately following acute exercise in WT animals, accompanied by an exercise-induced increase in the autophagosomal marker LC3II on isolated mitochondria, an effect that was abolished in the absence of Parkin. Thus, these results support a role for Parkin in mediating mitophagy during acute exercise.

Following 6 weeks of voluntary wheel training, Parkin localization to mitochondria was significantly enhanced, in correspondence with the observed increase in Parkin expression in trained muscle. However, we did not observe any downstream consequences of this translocation, such as an increase in basal mitophagic LC3II or p62 flux in trained WT animals. Instead, we speculate that this heightened expression and localization of Parkin to mitochondria may serve to “prime” mitophagy and increase the cellular potential for mitochondrial turnover in response to future bouts of exercise. To examine this possibility, a subgroup of trained animals was treated to an additional bout of exercise to determine the functionality of this adaptation. Our results indicate that trained animals respond to acute exercise with an attenuated mitophagy flux, compared to untrained animals. This attenuation is likely a result of reduced exercise stress signaling toward kinases that normally initiate mitophagy, in the presence of a training-induced enhancement of mitochondrial content and function [6, 72]. This would include reduced activation of AMPK, p38, and ROS signaling [73, 74], accompanied by greater endurance performance and a trend for reduced lactate production in muscle with a higher oxidative capacity. This was supported by a significant main effect of training on the attenuation of phosphorylated ACC, indicating reduced AMPK activation as a consequence of training-induced improvements in mitochondrial content.

To determine if Parkin is involved in mediating these beneficial adaptations, Parkin KO animals were also subjected to 6 weeks of endurance training. As with WT animals, endurance training had no impact on basal mitophagy flux in the absence of Parkin. However, unlike WT animals, the attenuation of acute exercise-induced mitophagy flux was not observed in KO animals following training. We also detected a greater reduction of phosphorylated ACC following training in the absence of Parkin, suggesting a lower transcriptional drive toward mitochondrial biogenesis, at least via AMPK. Previous research has also shown that exercise training can ameliorate mitochondrial dysfunction in muscle [6, 72, 75]. However, our data show that training was unable to improve mitochondrial respiration and attenuate ROS production in the absence of Parkin. Thus, these data indicate that Parkin is required to facilitate exercise-induced mitochondrial turnover and to confer mitophagy adaptations to endurance training. The data also support an important role for Parkin in mediating the accrual of functional mitochondria during adaptations to exercise training.

## Conclusions

Collectively, our study reveals several aspects of Parkin function in muscle with endurance exercise. First, Parkin is required for the maintenance of mitochondrial function as observed in KO animals that display both reduced mitochondrial respiration and enhanced ROS emission. Second, we found that endurance training did not alter basal mitophagy flux regardless of genotype. However, training resulted in an attenuation of exercise-induced mitophagy flux in WT animals, an effect that did not occur in the absence of Parkin. Additionally, mitophagy flux did not increase with acute exercise in KO animals, implicating Parkin’s requirement for mitochondrial turnover with exercise. This lack of response to exercise (and possibly other cellular stresses) in KO animals likely contributes to the accumulation of defective organelles, as documented here, in the absence of Parkin. Finally, mitochondrial content was enhanced following training in KO mice to the same degree as in WT animals, but this adaptation appeared to be PGC-1α-independent. This suggests that the absence of Parkin induces alternative signaling pathways that govern mitochondrial biogenesis in response to exercise. Even so, the training-induced biogenesis response led to mitochondria with persistent respiratory deficits and enhanced ROS emission in the KO animals. Thus, training did not rescue this mitochondrial dysfunction observed in the absence of Parkin, and this could explain why an attenuation in mitophagy by training was not observed, compared to WT animals.

## Abbreviations

ACC: Acetyl-CoA carboxylase
ADP: Adenosine diphosphate
AMPK: AMP-activated protein kinase
ATP: Adenosine triphosphate
Col: Colchicine
COX: Cytochrome c oxidase
IMF: Intermyofibrillar
KO: Knockout
LC3: Microtubule-associated proteins 1A/1B light chain 3B
mtDNA: Mitochondrial DNA
NIX: BCL2-interacting protein 3-like
NUGEMP: Nuclear genes encoding mitochondrial protein
OMM: Outer mitochondrial membrane
p62: Sequestosome-1
PARIS: Parkin-interacting substrate
PGC-1α: Peroxisome proliferator-activated receptor gamma coactivator 1-alpha
PINK1: PTEN-induced putative kinase 1
ROS: Reactive oxygen species
TFAM: Mitochondrial transcription factor A
Veh: Vehicle
VO_2_: Maximum rate of oxygen consumption
WT: Wild type
